# Targeted therapy in patients with genetic tumor syndromes

**DOI:** 10.1515/medgen-2025-2045

**Published:** 2025-11-08

**Authors:** Arne Jahn, Christoph Heining, Stefan Fröhling, Hanno Glimm, Evelin Schröck

**Affiliations:** University Hospital Carl Gustav Carus at TUD Dresden University of Technology and Faculty of Medicine of TUD Dresden University of Technology Institute for Clinical Genetics Fetscherstr. 74 01307 Dresden Germany; University Hospital Carl Gustav Carus at TU Dresden National Center for Tumor Diseases (NCT) Fetscherstr. 74 01307 Dresden Germany; German Cancer Research Center (DKFZ) Division of Translational Medical Oncology Im Neuenheimer Feld 280 69120 Heidelberg Germany; University Hospital Carl Gustav Carus at TU Dresden National Center for Tumor Diseases (NCT) Fetscherstr. 74 01307 Dresden Germany; University Hospital Carl Gustav Carus at TUD Dresden University of Technology and Faculty of Medicine of TUD Dresden University of Technology Institute for Clinical Genetics Fetscherstr. 74 01307 Dresden Germany

**Keywords:** Precision oncology, biomarker-targeted therapy, genetic testing and prevention, genetic tumor syndrome, clinical trials

## Abstract

Significant progress in comprehensive molecular diagnostics and targeted therapies for advanced malignancies has, in part, led to substantial improvements in patient outcomes. Nevertheless, comprehensive genomic profiling necessitates interdisciplinary discussion of potential clinical recommendations within interdisciplinary molecular tumor boards. (Likely) pathogenic germline variants (PGVs) typically warrant genetic counseling for patients and, where appropriate, their relatives. Concurrently, the rapidly expanding availability of targeted therapies introduces new therapeutic implications based on germline alterations that must be integrated into clinical decision-making. Moreover, the identification of PGVs may not only inform therapy in patients with manifest malignancy but also offer opportunities for targeted chemoprevention.

## Introduction

Increasing targeted therapy options are available and have significantly changed clinical management in many cancer entities. Targeted therapies shift towards earlier treatment lines and require molecular analysis that enable identification of genetic alterations or biomarkers with diagnostic (information on type of disease), prognostic (information on course of disease) and predictive (information on response to a specific therapy) relevance. In addition to somatic alterations, germline variants gain growing attention due to their increasing clinical actionability by novel targeted cancer therapies.

Precision oncology studies performing parallel tumor and normal tissue analyses have yielded a substantial number of monogenic (likely) pathogenic germline variants (PGVs) of 10–15% across cancer entities [29, 31]. This review aims to provide an overview of the therapeutic actionability of PGVs in cancer disposition genes and emphasizes the importance of multidisciplinary collaboration for improved patient care.

## Pathways how PGVs can be identified in a diagnostics and research context

**Figure 1:**
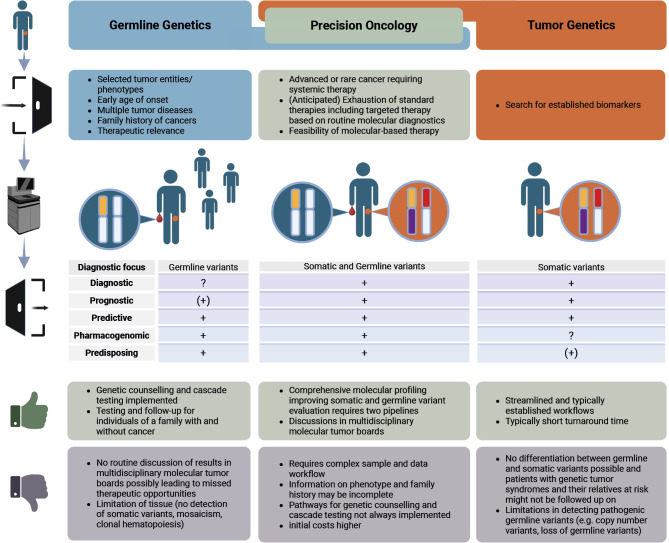
Pathways identifying somatic and/or germline alterations Created in BioRender. Jahn, A. (2025) https://BioRender.com/polh620

There are multiple pathways how patients with PGVs in cancer disposition genes can be indentified. It is primarily based on clinical or molecular prioritization before testing (Figure 1). Clinical parameters that should be obtained as part of the routine oncological work-up comprise for instance results of routine diagnostics and clinical investigation, history of multiple primary cancers with (immuno)histology and age of onset, family history of cancer, other diagnoses/abnormalities such as colon polyps or macrocephaly, and excessive toxicity. Check-lists are intended to confer with a clinical geneticist or to trigger genetic counseling. Recommendations are available, e.g. for children with cancer (e.g. DKG, https://www.krebsgesellschaft.de/zertdokumente.html), common cancer entities (e.g. DKG), and certain rare cancers (e.g. World Health Organization (WHO) Classification of Haematolymphoid Tumours [23] or NCCN Clinical Practice Guidelines in Oncology, Version 2.2025 Myelodysplastic Syndromes (NCCN Guidelines®)), or on minimal panels by ASCO [66] However, implementation of germline testing in clinical routine seems to be challenging for treating physicians, recommendations are lacking for many cancer types, and even further, the use of check-lists or inclusion criteria shows a limited sensitivity and misses about half of patients with PGVs [43].

In a diagnostic context, PGVs in cancer disposition genes can also be identified as an actionable secondary finding in the context of indication-based diagnostic clinical genetic testing. Different initiatives set up curations of clinical actionability, such as ClinGen based on assessing (1) severity of the outcome, (2) likelihood of the outcome, (3) effectiveness of the intervention in preventing or mitigating the outcome, and (4) nature (risk/burden) of the intervention [50]. The current minimum list of genes for opportunistic screening by the American College of Medical Genetics and Genomics contains 28 cancer disposition genes [45] and patients with PGVs in these genes showed a poorer survival [32].

Alternatively, there can be a rationale for germline testing to identify therapeutically relevant PGVs, or somatic testing can indicate a PGV in patients with advanced or metastatic (typically solid) tumors considered for such a treatment. This testing strategy is independent of criteria for genetic testing based on family history and clinical criteria used for familial risk assessment [10]. Testing is increasingly performed to identify clinically relevant germline and/or somatic variants in *BRCA1/2* in four cancer entities (Table 2) and to detect microsatellite instable tumors, possibly related to Lynch syndrome, which requires further workup. However, the number of genes to be tested for is rather limited which results in incomplete identification of patients with genetic tumor syndromes. If tumor only sequencing is performed, a screening for potential clinically relevant germline findings and further clinical genetic workup is recommended due to limitations in variant detection and assignment as germline variant [39].

In a personalized medicine setting and in a research context, molecular characterization typically comprises exome or genome sequencing, performed as paired tumor tissue and blood samples, as well as further molecular layers (RNA, DNA methylation and proteome), and results are discussed in a multidisciplinary molecular tumor board. Comprehensive molecular tumor profiling is typically offered to patients with advanced tumor disease [10]. Multiple programs and initiatives have been established in Germany, e.g. NCT/DKTK/DKFZ-MASTER with very broad molecular genome profiling for patients with rare cancers [27, 31], INFORM for children and young adolescents [25], CATCH/COGNITION for patients with breast cancer [26], comprehensive panel testing in the German network for personalized medicine (DNPM) [30], and nationwide panel sequencing for patients with non-small cell lung cancer in the National Network for Genomic Medicine [48].

As part of the multidisciplinary team, human geneticists must be included to evaluate (potential) germline variants [54]. For this purpose, comprehensive data collection and sharing with the human genetics team is required. Germline variant evaluation should be performed according to latest evidence based on increasingly gene specific criteria with specific population or in-silico prediction cut-offs and recommendations for the use of specific databases and studies for functional assessment by the ACMG/AMP, ClinGen or ACGS [53].

Certain limitations should be carefully considered during genetic testing. In the context of cancer patients, these include possible false positive results through hematologic malignancies or clonal hematopoiesis of intermediate potential, especially in elderly people treated with cytotoxic drugs. Alternatively, false negative results could result from allogenic hematopoietic stem cell transplantation, selection of normal tissue in case of post-zygotic mosaicism (see chapter mosaicism), alterations in genes so far not linked to disease and limitations of panel- and short-read sequencing to detect intronic and complex structural variants or altered methylation.

## Actionability of pathogenic germline variants

Diagnosis of a genetic tumor syndrome can have multiple impacts. In addition to psychosocial and socioeconomic effects, clinical actionability of PGVs variants can impact on:

### Therapeutic strategies

(I) Impact on conventional therapies such as cytotoxic drugs and radiation, e.g. in the case of heritable retinoblastoma affecting the risk for secondary cancer.
(II) (investigational/experimental) targeted therapies, in-label, off-label, on-study, compassionate use, described in the next section.

### Risk management

(I) Risk adjusted cancer surveillance or screening typically based on clinical assessment, imaging and biochemical screening, which has shown clinical benefit for patients with hereditary breast and ovarian cancer, Lynch syndrome and *TP53*-associated Li Fraumeni syndrome, and gains increasing relevance for long-term survivors due to improved therapies.
(II) Prevention of primary manifestations by life style, such as avoiding sun exposure for patients with *CDKN2A*-related tumor syndrome, by vaccinations such as HPV for patients with Fanconi anemia, or risk-reducing interventions, such as oophorectomy for patients with *BRCA1/2*-associated hereditary breast and ovarian cancer and (procto)colectomy for patients with familial adenomatous polyposis, or possibly chemoprevention such as through aspirin.
(III) Predictive testing and risk adjusted clinical management of relatives.
(IV) Potential prognostic impact, currently with limited clinical relevance [32].
(V) Reproductive planning.

Within this rapidly evolving field, clinical recommendations for patients with predominantly rare genetic tumor syndromes, such as from the European Reference Network ERN-Genturis (https://www.genturis.eu/l=por/guidelines-and-pathways/clinical-practice-guidelines.html) or NCCN Clinical Practice Guidelines in Oncology (NCCN Guidelines®) are valuable but must be regularly updated. Due to the limited availability of (experimental) targeted treatments for patients that exhausted established treatment recommendations and expert-level evidence for cancer surveillance for patients with genetic tumor syndromes, reimbursement has to be clarified.

**Table 1:** National Center for Tumor Diseases (NCT) and German Cancer Consortium (DKTK) Levels of evidence facilitating communication and discussion of molecular findings and their clinical relevance (Leichsenring *u.a.*, 2019 [41])

Data source	Evidence level	Description
Same tumor entity	m1A	In the same tumor entity, the predictive value of the biomarker or clinical efficacy was demonstrated in a biomarker-stratified cohort of an adequately powered prospective study or meta-analysis.
	m1B	In the same tumor entity, the predictive value of the biomarker or the clinical efficacy was demonstrated in a retrospective cohort or case-control study.
	m1C	One or more case reports in the same tumor entity.
Other tumor entity	m2A	In another tumor entity, the predictive value of the biomarker or clinical efficacy was demonstrated in a biomarker-stratified cohort of an adequately powered prospective study or meta-analysis.
	m2B	In another tumor entity, the predictive value of the biomarker or clinical efficacy was demonstrated in a retrospective cohort or case-control study.
	m2C	Regardless of the tumor entity, clinical efficacy was demonstrated in one or more case reports when the biomarker was present.
In vitro or animal model	m3	Preclinical data (in vitro/in vivo models, functional studies) show an association of the biomarker with the efficacy of the drug(s), which is supported by a scientific rationale.
Biological rationale	m4	A scientific, biological rationale suggests an association of the biomarker with the efficacy of the drug(s), which is not yet supported by (pre)clinical data.

## Targeted therapies in patients with genetic tumor syndromes

The standards of care and approvals of targeted therapies are rapidly evolving (see https://www.cancer.gov/about-cancer/treatment/types/targeted-therapies/approved-drug-list, https://www.ema.europa.eu/en/medicines). Increasingly, targeted therapies and immunotherapies are approved in an entity agnostic manner based on specific biomarkers, such as immune checkpoint inhibitors for cancers with high tumor mutational burden or microsatellite instability or tyrosine kinase inhibitors for malignancies driven by defined alterations of *BRAF*, *RET* or *NTRK* [63]. To allow prioritization of treatment recommendations beyond approved drugs, levels of predictive evidence for biomarkers have been categorized [41]. The NCT/DKTK levels of evidence demonstrate the molecular biomarker-drug association according to (i) tumor entity (i.e., histopathological classification), (ii) preclinical versus clinical evidence, and (iii) strength of clinical evidence regarding biomarker-driven trials or case reports [28] (Table 1). As an example, identification of a heterozygous PGV in *CHEK2* in a patient with a rare sarcoma could lead to a recommendation for a PARP inhibitor with an evidence level of m2B (based on evidence in prostate cancer, Table 2), especially when a somatic loss of heterozygosity and a single-base substitution signature 3 (SBS3, BRCAness) indicate a relevance of the *CHEK2* variant for the tumor.

According to this evidence scoring, recent literature reports that about half of all PGVs in cancer disposition genes are supporting molecularly stratified therapy recommendations in cancer patients [60, 31], which translates into approximal 4% of all patients for which an approved therapy could be suitable. Germline variants are by definition clonal so that targeting of germline variants could in theory be more effective compared to somatic and possibly clonal alterations. However, like somatic alterations, targeted therapies against germline variants can develop resistances (e.g. reverting germline variants). Like somatic variants, the main drug classes for patients with PGVs are small molecules and monoclonal antibodies [64]. It is challenging for clinicians (including human geneticists) to keep track of new approvals and it would be desirable that databases annotate therapeutic options based on PGVs as planned for the knowledge database OncoKB [61]. We provide an overview of selected established and investigational/experimental targeted therapies (Table 2).

The most common recommendations for targeted therapies are PARP inhibitors and immune checkpoint inhibitors, based on PGVs in homologous directed repairs, especially *BRCA1* and *BRCA2*, and Lynch syndrome-associated genes, respectively [60, 31, 12]. While PARP inhibitors are approved for *BRCA*-associated cancers (Table 2), their relevance in other entities is a subject of clinical investigation [34]. First clinical trials investigated PARP inhibitors in a tumor-agnostic design and confirmed previous reports of efficacy in *BRCA*-mutated uterine leiomyosarcomas [13, 57]. The predictive value of pathogenic variants in homologous recombination repair associated genes other than *BRCA1*/*2* has not yet been well explored beyond prostate cancer. However, case reports, such as demonstrating PARP inhibitor efficacy in a patient with a neuroblastoma and a PGV in *BARD1* [14], suggest potential clinical benefit. Therefore, in non-*BRCA* associated cancers a more comprehensive genomic profiling might be required to predict sensitivity to PARP inhibitors. To that end, the DKTK has completed a prospective clinical trial quantifying somatic and germline biomarkers as a compound biomarker (including SBS3 mutational signature, homologous recombination deficiency score and single nucleotide variants and indels in a predefined set of genes) to predict response to PARP inhibition and results are awaited (clinical trials.gov identifier NCT03127215). This score also includes heterozygous PGVs in genes associated with recessive disease (carriers), whose clinical relevance (especially diagnostic and therapeutic) is not well explored. As an example, emerging evidence suggests that a somatic second hit in carriers of PGVs in *MUTYH* can be oncogenic and might be clinically actionable [2].

**Table 2:** Selected experimental and approved targeted therapies partially based on PGVs (oncogenes: light blue and tumor suppressor genes: light red, sorted by approximated decreasing prevalence) with evidence of efficacy

Gene(s)/alteration	Entity/ phenotype	Mechanism of action	Drug	FDA appr.	EMA appr.	Selected evidence and results
*BRCA1/2*	HER2-negative metastasized breast cancer	PARP inhibitor	Olaparib Talazoparib	2018 2018	2019 2019	OlympiAD (adjuvant, phase III, n=302): olaparib vs. standard treatment (capecitabine, vinorelbine, or eribulin); PFS: 7.0 vs. 4.2 months (HR 0.58; 95% CI: 0.43 – 0.80); OS: 19.3 vs. 17.1 months (HR 0.90; 95% CI: 0.66 – 1.23) [55]
*BRCA1/2* (and e.g. ATM, BRIP1, BARD1, CHEK2, FANCL, PALB2, RAD51C, RAD51D*)	Metastatic castration-resistant prostate cancer	PARP inhibitor	Olaparib Rucaparib (Talazoparib)	2020 2020 2023	2020 - 2024	PROfound (phase III, n=387): olaparib vs. investigator’s choice of new hormonal agent (enzalutamide or abiraterone acetate), rPFS 9.8 vs. 3.0 months (HR 0.22 (95% CI 0.15, 0.32), OS 20.1 vs. 14.4 months (HR 0.63 (95% CI0.42, 0.95) [15]
*BRCA1/2*	Platinum sensitive metastatic pancreatic adenocarcinoma	PARP inhibitor	Olaparib	2019	2020	POLO (phase III, n=154 patients with no progression on platinum based first line therapy), olaparib vs. placebo, PFS 7.4 vs. 3.8 months (HR: 0.53 (95% CI 0.35–0.82)), OS: 19.0 vs. 19.2 months (HR: 0.83 (95% CI 0.56–1.22)) [20]
*BRCA1/2*	Platinum sensitive high-grade ovarian cancer	PARP inhibitor	Olaparib Rucaparib (Niraparib)	2014 2016 2017	2014 2018 2017	SOLO1 (first line maintenance, phase III, n=806): olaparib plus bevacizumab vs. placebo plus bevacizumab, mPFS 22.1 months vs. 16.6 months (HR for disease progression or death, 0.59; 95% CI 0.49 to 0.72) [52], OS: not reached vs. 41.9 months (HR 0.50 (95% CI 0.35–0.72) [16]
MSI-high TMB-high (Lynch syndrome, *POLD/E*?)	Solid tumors	checkpoint inhibitor (Anti-PD-1 antibody)	Pembrolizumab, Ipilimumab, Nivolumab	2017 2020	Entity specific	5 single arm clinical trials (total n=149), pembrolizumab, ORR 39.6% (95% CI: 31.7, 47.9), 11 complete responses and 48 partial responses [40] Immune checkpoint inhibitors for POLE or POLD1 proofreading-deficient metastatic colorectal cancer (retrospective, n=27), ORR compared to dMMR/MSI-H metastatic colorectal cancer 89% versus 54%, improved PFS (HR 0.17, 95% CI 0.04–0.69) and OS (HR 0.24, 95% CI 0.06–0.98) [1] and other entities [56]
*NF1*	(Pediatric) inoperable plexiform neurofibromas	MEK 1/2 inhibitors	Selumetinib, Mirdametinib	2020 2025	2018 2025	SPRINT (phase II, n=50 children): 68% with confirmed partial response, and 82% of these had a durable response (lasting ≥1 year) [21] ReNeu (phase IIb, n=58 adults and 56 children): ORR 41% of adults and 52% of children, median volumetric best response –41/42% [47] KOMET (phase III, n=145, randomised, placebo-controlled): ORR 20% vs. 5%, median response 3.7 months [11]
*TSC1/2*	TSC-associated angiofibroma, partial-onset seizures, subependymal giant cell astrocytoma, lymphangioleiomyomatosis and renal angiomyolipoma	mTOR Inhibitors	Sirolimus/ Everolimus	yes	yes	EXIST-1 (phase III, n=117), everolimus vs. placebo, subependymal giant cell astrocytoma response rate 34.6 vs. 0% [18] EXIST-2 (phase III, n=118), everolimus vs. placebo, angiomyolipoma response rate 41.8 vs. 0% [3] EXIST-3 (phase III, n=366), everolimus (two exposures) vs. placebo, response rate 28.2 vs. 15.1% [19]
*PTEN*	Breast cancer, vascular malformations	mTOR/AKT inhibitors	e.g. Sirolimus, Capivasertib	No	No	Two patients with breast cancer showed a durable response to capivasertib plus paclitaxel [37], response of two vascular malformations upon treatment with sirolimus [9], phase I (56 days) and II (6 months) of sirolimus indicated some evidence of improvement of symptoms (EEG improvements, regression of skin and gastrointestinal lesions ) [38, 59]
*NF2*	*NF2*-related schwannomatosis with progressive tumors	Tyrosine kinase inhibitor (incl. ALK)	Brigatinib	No	No	INTUITT-NF2: phase II basket design, n=40), brigatinib, radiographic response for target tumors 10% and for all tumors 23% after median follow-up of 10.4 months (greatest benefit meningiomas and nonvestibular schwannomas) [51]
*VHL*	Adult patients with von Hippel-Lindau (VHL) disease and tumors; renal cell carcinoma	HIF-2α inhibitor	Belzutifan	2021	2024	Belzutifan for Renal Cell Carcinoma in von Hippel–Lindau Disease (phase II, n=61), ORR 49% (95% CI 36–62) in VHL-associated renal cell carcinoma; ORR 63% in hemangioblastomas and pancreatic neuroendocrine tumors, duration of response >12 months for >50% of patients [33]
*PTCH1, SUFU*	Basal-cell nevus syndrome associated basal cell carcinoma	Hedgehog Pathway inhibitor	Vismodegib Sonidegib	2012 2015 (both basal cell carcinoma)	2013 2015 (both basal cell carcinoma)	Inhibition of the hedgehog pathway in patients with basal-cell nevus syndrome (phase II, n=41) vismodegib vs. placebo, reduced rate of new surgically eligible basal-cell carcinomas (two *vs* 34 per patient per year, 17% of patients tolerated vismodegib continuously for 36 months [62]
*RET*	(Medullary) thyroid cancer	Selective RET inhibitors	Selpercatinib, Pralsetinib	2024	2022	LIBRETTO-531 (phase III, n=291) first-line selpercatinib vs. physician’s choice (cabozantinib or vandetanib), PFS 86.8% vs. 65.7%, ORR 69.4% vs. 38.8%, duration of response not estimable vs. 16.56 months [22]
*PIK3CA*	Severe manifestations of PIK3CA-related overgrowth spectrum	PIK3CA inhibitor	Alpelisib	2022	2022, but withdrawn 2023	EPIK-P1 (retrospective, n=57) compassionate use alpelisib, 37.5% experienced a ≥20% reduction in target lesion(s) volume [8]
*EGFR*	Lung cancer	Kinase (EGFR) inhibitors	Osimertinib and others	Yes	Yes	INHERIT: Fourteen of 141 patients with the germline EGFR variant p.T790M received osimertinib, with potent activity and durable effect in the majority of patients (range appr. 3–60 months) [49]
*KIT/PDGRFA*	Gastrointestinal stromal tumors	Kinase (KIT/PDGRFA) inhibitors	Imatinib and others	Yes	Yes	Five case reports, whereof two with stable disease >= eight years [5]

PFS = progression-free survival, ORR = Objective response rate, CI = confidence interval, DDFS = distant disease free survival, IDFS = invasive disease free survival, OS = overall survival, rPFS = Radiological progression-free survival, n = number of patients included in the study, PGV = (likely) pathogenic germline variant, FDA = Food and Drug Administration, EMA = European Medicines Agency

* including (likely) pathogenic germline and/or (likely) oncogenic somatic variants

For patients with Lynch syndrome and typically MSI-high tumors, immune checkpoint inhibitors are a valuable treatment option and pathological complete responses have been reported. Besides established gene-based monotherapies, further treatment modalities such as combinations of targeted therapies, or combinations of targeted therapies with cytotoxic agents or radiation therapy hold the promise of clinical benefit for patients with genetic tumor syndromes. As an example, combination therapies such as the immune checkpoint inhibitor durvalumab combined with chemotherapy (carboplatin plus paclitaxel) in mismatch repair deficient endometrial cancers (NCT04269200) and the combination of two immune checkpoint inhibitors (nivolumab plus ipilimumab) for unresectable or metastatic MSI-high or mismatch-repair deficient colorectal cancer (NCT04008030) gained recent FDA approval.

Beyond PARP inhibition and immune checkpoint directed antibodies, other small molecules are available for patients with selected manifestations of several genetic tumor syndromes. In case of oncogenes, drugs can directly target mutated tyrosine kinases, either selectively (e.g. selective RET inhibitors) or multiple kinases (imatinib). Alternatively, upon inactivation of tumor suppressors, drugs can restore tumor suppressor function (e.g. belzutifan inhibiting HIF-2α in case of missing ubiquitylation due to PGVs in *VHL*), or indirectly target downstream signaling (e.g. mTOR inhibition due to PGVs in *TSC1/2* or MEK inhibition due to PGVs in *NF1*). In the future, the development of innovative variant specific inhibitors targeting frequent *TP53* and *KRAS* variants [58], or novel modalities such as cell-based, antisense nucleotide or gene replacement/editing therapies [42], being piloted in specific cancer entities or rare genetic diseases [24], might also prove valuable for patients genetic tumor syndromes. Limitations of clinical studies based on germline variants include their predominant focus on *BRCA1/2* alterations and PARP inhibitor therapies, the less frequent use of survival endpoints compared to non-germline trials [35], as well as limited case numbers and insufficient information on selection biases. These limitations challenge the refinement of positive and negative predictive values of potential clinical and demographic confounders such as race and ethnicity.

## Prevention strategies and further opportunities based on germline testing

In addition to treatment of cancers or manifestations of genetic tumor syndromes, (chemo)prevention strategies based on early identification of such syndromes are an emerging and promising area of research. An exciting example of a preventive therapy is the use of aspirin against polyps in patients with Lynch syndrome. The CAPP2 trial reported a hazard ratio of 0.65 for colorectal cancer in patients with at least 2 years of 600 mg aspirin daily vs. placebo [6] during a mean follow-up of ten years. A follow-up study (CAPP-3) investigated different doses of aspirin and results are expected soon. As another example, immune modulation in patients with Lynch syndrome might offer great opportunities for cancer prevention. While first retrospective data indicated that immune checkpoint inhibitor treatment might have a moderate effect on cancer risk, further strategies such as tumor vaccines are being clinically tested [4]. For women with pathogenic *BRCA1/2* germline variants there is limited and partially conflicting evidence of primary chemoprevention of breast and ovarian cancer with selective estrogen receptor modulators or aromatase inhibitors (NCCN Guidelines Version 1.2026. Genetic/Familial High-Risk Assessment: Breast, Ovarian, Pancreatic, and Prostate). Chemoprevention against cancers or other manifestations of rare genetic tumor syndromes are explored in early clinical trials. One example is a phase 1 study in children with tuberous sclerosis younger than six months indicating that sirolimus (mTOR-inhibitor) was well tolerated and had a favorable efficacy profile (seizures and intellectual development) (NCT04595513 and NCT05104983). Another example is the preventive use of metformin in addition to MRI surveillance in a randomized phase II trial for individuals with Li Fraumeni syndrome [17].

Apart from monogenic cancer syndromes, genetic germline testing might have further clinical impact in the future. Firstly, polygenic risk scores could be used to inform about cancer risk and to justify preventive measures (e.g. lifestyle adjustments), which could inform cancer surveillance or screening in a higher genetic risk group for certain cancer entities [7]. Secondly, germline testing comprises results on pharmacogenomics and predict drug efficacy or toxicity, which is important for an increasing number of drugs [65]. Thirdly, germline variants might have an underappreciated prognostic relevance, as recently reported for a common *PCSK9* germline variant associated with metastatic disease and reduced survival in a homozygous state in patients with breast cancer. Interestingly, *PCSK9*-targeted therapy (evolocumab), approved to treat hypercholesterolaemia, revealed a decreased metastatic potential of cancer xenografts in mice and organoids [44], indicating preventive potential for humans.

## Outlook

Due to an increasing clinical actionability of germline variants for the index patient and relatives, we foresee a raising demand for comprehensive germline testing. This will implicate certain challenges. Better integration of somatic and germline testing into routine care is required and could be implemented as an oncology care delivery model initiated promptly at cancer diagnosis, considering drug testing and drug approvals at earlier cancer stages [66]. This strategy has been successfully established in precision oncology studies [46]. However, certain barriers must be addressed. Referral and access to genetic testing are underutilized, counseling capacities have to be expanded and streamlined. Functional testing is necessary to improve variant (of uncertain significance) classification. Upon diagnosis, pathways for genetic counselling, therapeutic evaluation, possibly study inclusion and documentation in clinical records must be improved. Personalized drug developments, further clinical trials including germline variants (currently only 1.1% [35]) with innovative trial designs considering the rarity of most diseases (e.g. basket or n-of-1), as well as comprehensive data and follow-up collection, smart usage and evaluation of cost efficiency [36], are required to realize the promise for patients with genetic tumor syndromes.

## Abbreviations/Glossary

PGV: (likely) pathogenic germline variant
PFS: progression-free survival
ORR: Objective response rate
CI: confidence interval
DDFS: distant disease free survival
IDFS: invasive disease free survival
OS: overall survival
rPFS: Radiological progression-free survival
n: number of patients included in the study
MSI: microsatellite instability
